# A conceptual multi-agent architecture for mental health triage in post-conflict Arabic-speaking populations: a theoretical proposition and staged validation argument

**DOI:** 10.3389/fpsyt.2026.1854701

**Published:** 2026-07-22

**Authors:** Anas Shahin, Bahaa Masry

**Affiliations:** 1 Faculty of Information Technology, Syrian Virtual University, Damascus, Syria; 2 Faculty of Medicine, Damascus University, Damascus, Syria

**Keywords:** Arabic NLP, cultural adaptation, HITL governance, mental health, mhGAP, multi-agent systems, post-conflict, Syria

## Abstract

Syria’s protracted conflict has produced a mental health crisis of extraordinary scale, with post-traumatic stress, depression, and anxiety estimated at several times global baselines, set against fewer than 0.37 psychiatrists per 100,000 people. Existing AI mental health tools have been developed and evaluated primarily for English-speaking, non-humanitarian populations, and their transfer to this setting is constrained by three simultaneous structural deficiencies—extreme clinical scarcity, Arabic natural-language-processing underperformance for dialect, and cultural misalignment with Syrian idioms of distress—which we term the *Triple Gap*. This article is a conceptual contribution in the Hypothesis and Theory genre, and its central claim is theoretical rather than technical: that AI-assisted mental-health triage at a safety floor adequate for crisis relevant care in this setting is conditional on the joint satisfaction of three constraints—linguistic adequacy for the local dialect, cultural validity for local idioms of distress and help-seeking, and bounded clinical responsibility through human oversight. These constraints interact, so that a system satisfying fewer than all three is expected to fail in clinically consequential rather than random ways. As one possible design response to this proposition—neither the only one nor a validated one—we describe a conceptual multi-agent architecture aligned with the WHO mhGAP task-shifting model: a four-stage pipeline (screening, risk stratification, routing, follow-up) constrained by a cross-cutting cultural-adaptation layer, augmented by candidate verification mechanisms with explicit abstention, and governed by human oversight in which clinical responsibility rests with a licensed clinician. The proposal is a hybrid clinical decision support hypothesis, not an autonomous system. Because no Syrian Arabic clinical corpus yet exists, the conversational components of the design cannot presently be evaluated; we therefore set out a staged sequencing argument for future work in which the construction of a Syrian Arabic Mental Health Evaluation Corpus (SAMHEC) is the first and rate-limiting condition. We present no prototype, no corpus, and no clinical, cultural, or safety evaluation, and we make no claim of clinical validity, safety, or readiness for deployment. The contribution is the integration of multi-agent triage with task-shifting and cultural adaptation into a single conditional argument whose adequacy can be established only through the staged empirical work we describe.

## 1 Introduction

### 1.1 The Syrian mental health crisis

The Syrian conflict, now in its fourteenth year, has produced one of the most severe mental health emergencies in contemporary humanitarian history. Epidemiological surveys consistently report posttraumatic stress disorder (PTSD) prevalence of 23–43%, major depression of 30–51%, and generalised anxiety of 18–49% amongst conflict-exposed populations (1–3). This burden is compounded by near-total collapse of psychiatric infrastructure: Syria has 0.37 psychiatrists per 100,000 people nationally, whilst Northwest Syria—home to nearly four million people—relies on just two practicing psychiatrists (4, 5). Mental health services in Northwest Syria are sustained by NGOs operating under severe constraints, with the WHO mhGAP Intervention Guide (6) serving as the standard for task-shifting to non-specialist community health workers (CHWs). Bou-Orm et al. (2) documented the operational challenges facing MHPSS services in this fragmented geography.

AI-powered mental health interventions have attracted growing attention as instruments of scale (7, 8). Multi-agent architectures, in which specialised LLM-based agents collaborate on complex clinical tasks, have shown improved assessment accuracy over single-model approaches on English-language benchmark datasets (9–11); we are explicit that this evidence concerns benchmark performance and not clinical deployment outcomes, and certainly not deployment in humanitarian settings. Direct transfer of these tools to Arabic-speaking, post-conflict settings is structurally constrained rather than already demonstrated to fail: Arabic NLP lags behind English in clinical domains (12, 13), Syrian dialect is nearly absent from NLP benchmarks (14), and the ethical terrain of algorithmic deployment in fragile contexts is undertheorised (15). To our knowledge, no peer-reviewed deployment of multi-agent mental health AI in Syrian Arabic-speaking populations has been published; this absence—rather than a demonstrated failure—is the starting point of the present analysis.

Throughout, we use mental health triage in a deliberately narrow sense: an initial-contact decision-support task that detects symptom-relevant signals from a user-initiated or community-health-worker–mediated conversation, assigns a four-tier severity classification (mild, moderate, severe, crisis), recommends a referral destination aligned with mhGAP tiered care, and supports longitudinal re-contact for deterioration monitoring. Triage in this sense is consultative rather than diagnostic: it produces a recommendation that a human supervisor accepts, modifies, or overrides, and the boundary of clinical responsibility rests with that supervisor rather than with the system, which makes no clinical diagnosis.

Because the design we develop is hybrid clinical decision-support rather than an autonomous system, the supervising human is constitutive of the workflow rather than an external safeguard. Two supervisory roles recur throughout the argument: an mhGAP-trained community health worker, who reviews moderate-tier routing and a daily mild-tier digest under a standing clinical protocol authored by a licensed clinician; and a licensed clinician (psychologist, psychiatrist, or general practitioner with mental-health training), who reviews all severe- and crisis-tier recommendations and all abstention outputs before any care action is taken, and with whom clinical responsibility and liability rest. The system is a recommender, not a decision-maker; the full responsibility model is developed in Section 2.5.

### 1.2 Arabic NLP for mental health

Arabic remains critically underrepresented in clinical NLP. Alasmari (12) identified the lack of domain-specific Arabic datasets as the primary barrier, noting that transformer-based models pretrained on Arabic corpora—AraBERT, MARBERT, and CAMeL—outperform classical approaches but trail English equivalents substantially. Fouda et al. (13) evaluated eight LLMs on Arabic mental health datasets, finding that few-shot prompting improved GPT-4o Mini accuracy by a factor of 1.58 on multi-class classification, whilst model selection proved more consequential than language configuration. Al-Khateeb et al. (14) documented ongoing challenges in adapting LLMs for Arabic healthcare communication, particularly for dialectal variation, and multilingual alignment work highlights code-switching as an additional evaluation concern for LLMs (16). Syrian Arabic specifically remains near-absent from publicly available NLP benchmarks. The consequence for any system in this setting is direct: dialect-aware processing cannot be treated as an available capability but must be built and benchmarked before downstream claims can be evaluated. This is why linguistic adequacy enters the proposition developed below as the first of its three constraints, and why the construction of a dialect corpus becomes the rate-limiting step of any serious validation effort.

### 1.3 Multi-agent AI for mental health

The emergence of LLM-based multi-agent systems has created a new paradigm for clinical AI. Hu et al. (9) proposed AgentMental, featuring specialised agents for questioning, adequacy evaluation, scoring, and adaptive follow-up, validated on the DAIC-WOZ dataset. Lee et al. (11) demonstrated that multi-agent debate improves clinical reasoning in personalised mental health scenarios. Bi et al. (10) showed structured multi-agent interview protocols outperform single-agent approaches on psychiatric benchmarks, whilst Kim et al. (17) introduced adaptive LLM collaboration for medical decision-making. A retrieval-augmented multi-agent framework by Xiao et al. (18) achieved 12.3% higher diagnostic accuracy than baseline GPT-4o on 561 real clinical cases. Alhuzali et al. (19) evaluated pretrained language models for Arabic mental health Q&A classification, establishing initial benchmarks for the Arabic domain. Beyond mental health proper, the closest humanitarian analogue is EMPATHIA (20), a three-agent (emotional, cultural, ethical) selector–validator framework for refugee placement decisions, which shares our concern with culturally grounded multi-perspective AI in fragile-state contexts but addresses an allocation rather than a clinical-triage task. Theorem-of-Thought (21) further demonstrates that parallel reasoning agents with cross-checking outperform strictly feed-forward chains on reasoning benchmarks, a finding that informs our verification design choices in Section 2.3.6.

What remains absent is any framework designed for Arabic-speaking, post-conflict populations that integrates cultural adaptation, humanitarian care alignment, and ethical governance for fragile-state deployment. Two features of this body of work shape the design we propose. Multi-agent decomposition with cross-checking has outperformed single-model approaches on the assessment-style tasks closest to ours, which is the empirical reason to prefer a decomposed over a monolithic design; but none of these results was obtained on Arabic-language or fragile-state data, so the decomposition and verification strategies we adopt are informed by this evidence without being licensed by it, and their adequacy on a Syrian Arabic clinical distribution remains an open question rather than an established result.

### 1.4 AI ethics in humanitarian contexts

The WHO (15) has issued governance guidance for LLMs in healthcare, calling for transparency, rigorous evaluation, and oversight proportionate to risk. Obradovich et al. (22) catalogued opportunities and risks of LLMs in psychiatry, emphasizing that deployment in fragile contexts amplifies data privacy risks, algorithmic bias from non-representative training data, and accountability gaps. Fitzpatrick et al. (23) and Inkster et al. (24) demonstrated the clinical potential of conversational AI agents for mental health in English-language populations, while underscoring the limitations of such tools when applied outside their original design contexts. This governance literature converges on a requirement that is institutional as much as technical: deployment in a fragile setting must rest on bounded clinical responsibility, with explicit decision authority and liability allocation rather than diffuse oversight, and the same literature cautions that clinical decision-support in psychiatry is not, as a class, currently ready for routine deployment (25, 26). These considerations supply the third constraint of the proposition that follows, and the reason the design is consultative rather than autonomous and treats comparative-effectiveness testing as a distant rather than a near-term prospect.

### 1.5 Study aims and contributions

The theoretical core of this article is a single proposition about the conditions under which AI-assisted mental health triage can be made safe in fragile-state Arabic-speaking populations:

*In post-conflict Arabic-speaking populations, AI-assisted mental health triage at the safety floor required for crisis-relevant care is conditional on the joint satisfaction of three constraints — linguistic adequacy for the local dialect, cultural validity for local idioms of distress and help-seeking, and bounded clinical responsibility through human oversight. Any one of these constraints, addressed in isolation, is insufficient; the constraints interact, and a system that satisfies fewer than all three is predicted to fail in clinically consequential ways.*

This is a claim about the conditions of adequacy for triage systems in this setting; it does not by itself specify a number of agents or any particular verification mechanism. The Triple Gap (Section 2.2) provides the explanatory account of why these three constraints arise together in this context, and the architecture developed in Section 2.3 is one candidate design that translates the proposition into a buildable specification. As a subordinate and testable conjecture, we advance that a triage workflow decomposed into four specialised stages — screening, risk stratification, routing, and follow-up — constrained by a cultural-adaptation layer, augmented by verification with explicit abstention, and supervised by a trained human at the severe and crisis threshold, is one reasonable response to the proposition. Whether it is an adequate response is what the staged evaluation in Section 4 is designed to test, against criteria such as triage concordance with mhGAP-trained community health workers, a high crisis-detection sensitivity floor, and reduced inappropriate routing relative to a non-adapted single-model comparator; these are properties of the candidate design rather than of the proposition itself, and we state them qualitatively here, leaving their provisional numerical form to the roadmap.

The choice of a decomposed rather than monolithic design follows from the structure of the task and its failure modes, not from a preference for architectural complexity. The mhGAP intake-to-disposition workflow is itself sequential — screening, severity assessment, referral, follow-up — so a single classifier cannot represent the routing and follow-up phases without auxiliary logic; the characteristic failure modes of these phases differ in kind (dialect-driven misclassification, threshold miscalibration, registry staleness, loss of contact), so locating each at a distinct stage makes graceful degradation and abstention tractable where a monolithic model would degrade opaquely; and the verification we describe presupposes intermediate, inspectable outputs at the high-stakes stage. None of this shows that four stages is optimal — a three- or five-stage decomposition could satisfy the same considerations — only that some non-trivial decomposition is warranted, and we treat the precise number as open to revision in light of evidence.

The contribution of the article is correspondingly conceptual, and each of its components makes a different kind of claim. The Triple Gap is an interpretive lens, drawing together health-system capacity arguments, the sociotechnical model of health information technology (27), and cross-cultural psychiatry (28) to explain why solutions developed elsewhere are unlikely to transfer directly; it is a framework rather than a tested causal model. The architecture is a design illustration of how the proposition might be satisfied, presented with no implemented component and no clinical evaluation. The comparative mapping (Section 3) is a preliminary, non-systematic positioning of this design against published multi-agent mental-health frameworks, not a demonstration of superiority. And the validation argument (Section 4) sets out the sequence of evidentiary conditions — beginning with corpus construction — that would have to be met before any of these claims could be tested. No component has been implemented, no Syrian Arabic corpus collected, and no clinical, cultural, or safety evaluation conducted; the standing of the work is a conditional conceptual hypothesis throughout.

## 2 Methods

### 2.1 Method for constructing the framework

This section describes how the conceptual framework was constructed and makes that procedure explicit, because the article proposes a design rather than reporting an empirical study, and the credibility of a design proposal rests on the traceability of its requirements to source literature and operational constraints.

The use-case was bounded to first-contact mental health triage, in the operational sense given in Section 1.1, for adult Arabic-speaking individuals in post-conflict settings, reached either through self-initiated contact or through a community health worker conducting a structured intake under the WHO mhGAP Intervention Guide (6); paediatric triage, inpatient assessment, pharmacological decision-making, and diagnostic confirmation were placed out of scope, a bounding informed by the documented mental health workforce density in Northwest Syria (4, 5) and by operational reports from MHPSS organisations active in the region (2). Within this scope, requirements for the design were drawn from four bodies of literature: the mhGAP Intervention Guide and humanitarian MHPSS reports (2, 6), which supply the task-shifting reference model; Arabic clinical NLP (12–14, 19), which delimits achievable linguistic capability and its current limits; the cross-cultural psychiatry of conflict-affected Syrian populations (28), which defines the cultural-adaptation requirements; and AI ethics and governance guidance for healthcare and fragile-state contexts (15, 22, 25, 26). Each requirement is traceable to one or more of these sources through the citations carried into the design description that follows.

The four-stage decomposition follows the structure of the mhGAP intake-to-disposition workflow (screening, severity assessment, referral, follow-up) and is informed by recent multi-agent clinical AI (9–11, 17, 18). The cross-cutting cultural-adaptation layer is a second constraint on that decomposition, since the staged structure alone is task-structural and does not encode the linguistic and idiomatic requirements; human oversight and the candidate verification mechanisms (Section 2.3.6) are a third, responding to the high crisis-sensitivity floor and to documented limitations of model outputs (21, 29–31). The result is a literature-informed design synthesis rather than the product of formal requirements engineering or systematic case analysis, and we do not claim it is the unique design that satisfies these constraints. We note the limits of this procedure plainly: requirement extraction reflects the authors’ reading rather than a multi-rater coding protocol, the resulting design is one of several that could satisfy the constraints, and no consultation with practising MHPSS clinicians in Northwest Syria has yet been undertaken, a step we regard as necessary before any implementation work.

The comparison in Section 3 rests on a structured but non-systematic search. Existing multi-agent mental health frameworks were identified in February–March 2026 across Google Scholar, Semantic Scholar, ACL Anthology, and IEEE Xplore, using the search strings:

- (“multi-agent” OR “multi agent”) AND (“LLM” OR “large language model” OR “language model”) AND (“mental health” OR “psychiatric” OR “depression” OR “anxiety” OR “triage”), restricted to 2023–2025;
- (“agent-based” OR “agentic”) AND (“clinical decision support” OR “mental health screening”) AND (“Arabic” OR “humanitarian” OR “refugee”), unrestricted by year, used to surface humanitarian analogues.

supplemented by reverse-citation tracing from each identified framework. We included works with an explicit multi-agent or agentic architecture, a mental-health or directly adjacent humanitarian application, and a peer-reviewed or full-preprint methodological description, and excluded single-agent role-prompting systems, non-mental-health clinical domains, and demonstration-only reports without methodological detail; excluded titles are not enumerated, which is itself a limitation of the procedure. EMPATHIA (20) was retained on substantive grounds, as the only published multi-agent system operationalising culturally-grounded multi-perspective AI for a fragile-state population (Kakuma refugee camp) and thus the only available reference point for the cultural-adaptation and humanitarian-deployment dimensions of the comparison. No PRISMA registration was undertaken and full coverage is not claimed; feature coding (**Tables 1**, **2**) was performed by the first author, independently reviewed by the second, and disagreements were resolved against the primary sources.

**Table 1:** Design requirements for the proposed Syrian-Arabic post-conflict triage system, with the coding criterion used in **Table 2**.

Design requirement	Coding criterion (operational rule)	This Work
Multi-agent architecture	Pipeline of ≥ 2 specialised agents with typed inter-agent communication	✓^C^
Mental-health task	Output is a mental-health-relevant classification, screening signal, or triage recommendation	✓^C^
Arabic-language support	At least one model component fine-tuned or evaluated on Arabic-language clinical input	✓^C^
Dialect-aware NLP	Cited work explicitly addresses non-MSA dialectal variation in input or evaluation	✓^C^
Cultural adaptation	A documented design step in which culturally-specific idioms, stigma framings, or coping concepts inform the architecture beyond translation	✓^C^
Post-conflict/fragile-state design	Documented design step accounting for surveillance risk, displacement, intermittent connectivity, or shared device scenarios	✓^C^
HITL governance	Documented human review of severe/high-stakes outputs before action, with override and audit; “Partial^C^” indicates the work describes oversight in design but does not specify its operational form	✓^C^
Triage and routing	Output maps directly to a specific care-delivery destination, not only to a severity score	✓^C^
Longitudinal follow-up	System re-contacts the same user at ≥ 1 later time point, with deterioration detection	✓^C^
Verifier/cross-check	Mechanism that resamples or independently checks an agent’s output before downstream consumption	✓^C^
Hallucination handling	Mechanism that screens or suppresses model-generated user-facing content for fabrication	✓^C^
Abstention as output	Agent can refuse to commit to a typed output, with abstention logged as its own outcome category	✓^C^
Explainability	Agent generates a natural-language justification with at least one mechanism for faithfulness assessment	SHAP + faithfulness check^C^
mhGAP alignment	System architecture is mapped onto WHO mhGAP task-shifting tiers	✓^C^
Privacy architecture	Documented threat model beyond standard data protection, including surveillance-exposure considerations	Fragile-state^C^
Target population	Population for which the system is designed and evaluated	Syrian Arabic-speaking, post-conflict
Clinical instruments	Validated instruments used at the screening or stratification step	PCL-5/PHQ-9/GAD-7^C^ (V1 = structured admin.)

Each requirement is a property the future system would need to satisfy; each criterion is the operational rule used to code whether a cited work addresses that requirement. All entries for the present work are ✓^C^ (conceptual proposal only); no implementation, evaluation, or validation has been undertaken.

**Table 2:** Evidentiary maturity of five comparable multi-agent mental-health frameworks against the design requirements of **Table 1**.

Design requirement	(9)	(11)	(17)	(10)	(18)
Multi-agent architecture	✓^V^	✓^V^	✓^V^	✓^V^	✓^V^
Mental-health task	✓^V^	✓^V^	✓^V^	✓^V^	✓^V^
Arabic-language support	—	—	—	—	—
Dialect-aware NLP	—	—	—	—	—
Cultural adaptation	—	—	—	—	—
Post-conflict/fragile-state design	—	—	—	—	—
HITL governance	Partial^C^	—	✓^I^	Partial^C^	Partial^C^
Triage and routing	—	—	—	—	—
Longitudinal follow-up	—	—	—	—	—
Verifier/cross-check	—	Debate^V^	—	—	—
Hallucination handling	—	—	—	—	—
Abstention as output	—	—	—	—	—
Explainability	—	Debate^V^	—	—	—
mhGAP alignment	—	—	—	—	—
Privacy architecture	Standard	Standard	Standard	Standard	Standard
Evaluation evidence base	DAIC-WOZ^V^	Simulation^V^	Clinical^V^	Benchmark^V^	561 cases^V^
Target population	English	English	English	English	Chinese
Clinical instruments	PHQ-8	—	—	PHQ-8	13 scales

Cell states: ✓^C^ = conceptually described in cited work; ✓^I^ = implemented in a documented prototype; ✓^V^ = independently evaluated against a held-out benchmark or human reference standard; — = absent though within scope; N/S = not in scope. The comparator is the published literature on each system, not its design potential; readers should interpret the table as a snapshot of present evidentiary state, not a permanent property of any system.

The validation argument in Section 4 was specified by asking, for each part of the design, what would have to be observed for it to fail, and by ordering those tests according to the dependencies among them, so that corpus construction precedes component evaluation, which precedes integrated simulation, which precedes any field study. The numerical values that appear in that section are planning assumptions rather than pre-specified targets, and are identified as such where they occur.

The mapping from each requirement to the design element that addresses it, together with the source literature from which the requirement was drawn, is set out in **Table 3**, which makes the reasoning checkable against the cited primary sources.

**Table 3:** Traceability from source literature to design requirements to design decisions.

Source literature	Design requirement	Architectural decision
mhGAP Intervention Guide (6); MHPSS operational reports (2, 4)	Task-shifting workflow with screening → severity assessment → referral → followup	Four-agent decomposition mirroring the mhGAP intake-to-disposition phases (Agents 1–4)
Arabic clinical NLP (12–14, 19)	Dialect-aware processing of Syrian/Levantine Arabic and codeswitching, with documented performance gaps relative to English	Dialect adaptation component of the Cultural Adaptation Layer; Phase 1 codeswitching evaluation track; tier-conditional dialect-robustness target
Cross-cultural psychiatry (28); idioms of distress literature	Recognise somatic and religiously-inflected idioms as constitutive signal, not accessory features; avoid stigmatising clinical labels in user-facing output	Stigma-sensitive language and religious/cultural coping components of the Cultural Adaptation Layer
LLM hallucination and reliability literature (21, 29, 30)	Pure feed-forward pipelines insufficient at a high crisis-sensitivity floor; outputs may be fluent yet unsound	Transversal verification layer (semantic consistency cross-check; SAE-style feature monitor); abstention as a first-class output (31)
WHO governance guidance (15); LLMs in psychiatry (22)	Transparency, evaluation, and oversight proportionate to risk; non-deployment-ready status of decision-support (25, 26)	Human-in-the-loop governance with two-role responsibility model (Section 2.5); explicit consultative status; audit logging
Fragile-state surveillance and privacy literature; crosscultural psychiatry (28)	Stigma-aware help-seeking; surveillance exposure threat model; longitudinal recontact across unstable conditions	Pseudonymisation/anonymisation separation; partner-NGO custody of linkage keys; anonymous one-shot screening option; SMS/low-bandwidth fallback
SAMHEC pre-requisite (this work)	A Syrian Arabic mental-health corpus is required before any conversational free-text symptom extraction can be safely deployed	Phased rollout: Version 1 uses structured instruments (PHQ-9, GAD-7 Arabic translations); Version 2 conversational extraction contingent on Phase 1 validation

Each row reads: a source body of literature yields a specific design requirement, which is satisfied by a specific element of the proposed design.

### 2.2 The triple gap framework

We propose the Triple Gap as a diagnostic conceptual lens—not a predictive model or a closed analytical taxonomy—for understanding why existing solutions, both clinical and technological, are inadequate in the Syrian post-conflict mental-health context. The lens has explanatory force in three specific senses, which we set out before describing the three gaps individually.

First, the three gaps are not independent. The lens treats clinical scarcity, linguistic underperformance, and cultural misalignment as interacting structural conditions, not parallel obstacles. Clinical scarcity substantially increases reliance on non-specialist task-shifting (mhGAP), which in turn forces reliance on screening instruments and triage protocols; the linguistic gap then determines whether those instruments can be administered at all in dialect; and the cultural gap determines whether the symptom signals so elicited will be clinically meaningful. Closing any one gap without the others either fails on its own terms (a culturally adapted tool with poor Arabic NLP misclassifies; a high-accuracy Arabic NLP model without cultural adaptation produces stigmatising or dismissive outputs) or shifts failure to the next gap (better dialect NLP without specialist scarcity-aware routing produces accurate assessments with no downstream care pathway). This non-independence constitutes the principal analytical claim of the lens: it hypothesises that interventions addressing fewer than all three dimensions may exhibit recurrent and partially structured failure patterns rather than entirely isolated or random forms of breakdown.

Second, the lens integrates three established traditions whose interaction is not standard in the AI literature. It draws on health-system capacity arguments, in which mental health workforce density is treated as a structural determinant of access (4, 6) — the WHO mhGAP task-shifting model is itself a response to capacity shortfalls. It draws on the sociotechnical model of health information technology (27), in which technology, workflow, people, and external rules and culture form interdependent dimensions whose misalignment predicts intervention failure; the Triple Gap reads existing AI mental health tools as misaligned along the technology–people and technology–culture axes when transferred to Syrian dialect, idiom, and care infrastructure. It draws on cross-cultural psychiatry (28), in which idioms of distress, religious framings, and stigma are recognised not as accessory features but as constitutive of how clinical signal presents in non-Western settings. The contribution of the lens is not to introduce these three traditions, each well-established in its own field, but to make their joint application a precondition for AI design in the post-conflict Arabic-speaking case.

Third, the lens supports a falsifiable prediction. The Triple Gap predicts that AI mental-health interventions transferred to this context without joint cultural, linguistic, and capacity-aware adaptation will exhibit one or more of three specific failure modes: (i) low concordance with mhGAP-trained CHW judgement on culturally-framed presentations (cultural gap); (ii) systematic misclassification of dialectal and codeswitched input (linguistic gap); (iii) routing decisions whose appropriateness collapses under realistic specialist scarcity (capacity gap). These predictions are testable in principle by parallel administration of an existing English-trained system against an mhGAP CHW on the same Syrian Arabic-speaking caseload, and we identify this as a study a future research group could conduct independent of the present architecture. We do not claim that the lens is exhaustive, mutually exclusive, or stable over time; we claim only that it has the analytical content described above and may provide a useful interpretive framework for the preliminary design analysis of future systems.

**Figure 1:**
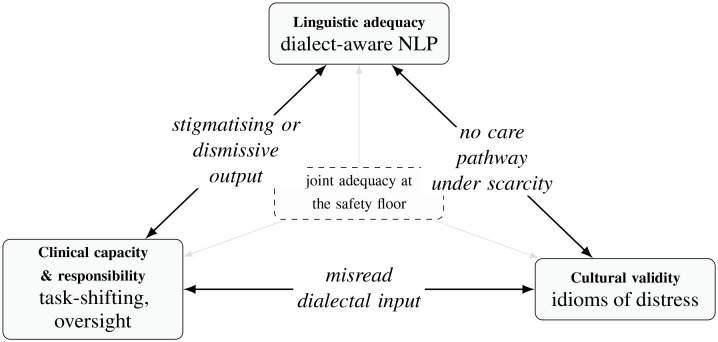
The triple gap: three interacting adequacy constraints for AI-assisted mental-health triage in post-conflict Arabic-speaking populations. The clinical, linguistic, and cultural constraints are shown with bidirectional pairwise interaction arrows; each interaction edge is labelled with the characteristic failure mode predicted when only one of the two constraints is satisfied. The dashed central element “joint adequacy at the safety floor” depends on all three constraints. Status: conceptual lens, not a tested causal model.

The three gaps are described separately for clarity below but should be read as interacting and amplifying one another. **Figure 1** illustrates the three structural failures and their interactions.

Clinical Gap. Syria’s specialist-to-population ratio [0.37 per 100,000 nationally; 0.05 per 100,000 in Northwest Syria (4)] makes individual clinical care structurally impossible at scale. Task-shifting through mhGAP (6) extends capacity but cannot close the gap without technological augmentation.

Technological Gap. Existing AI mental health tools are trained predominantly on English-language datasets from Western populations. Arabic NLP for mental health is critically underdeveloped: Fouda et al. (13) showed zero-shot LLM performance on Arabic mental health tasks remains markedly below English equivalents. Syrian dialect is absent from standard benchmarks, meaning off-the-shelf deployment risks systematically inaccurate assessments.

Cultural Gap. Distress in Syrian Arabic is expressed through somatic complaints, religiously-inflected idioms, and concepts such as *ma’anat* (suffering) that do not map to DSM/ICD categories (28). Stigma around mental illness (*junun* = madness) is substantial. An AI tool that ignores these dimensions will be rejected, underused, or harmful.

### 2.3 Proposed framework architecture

**Figure 2:**
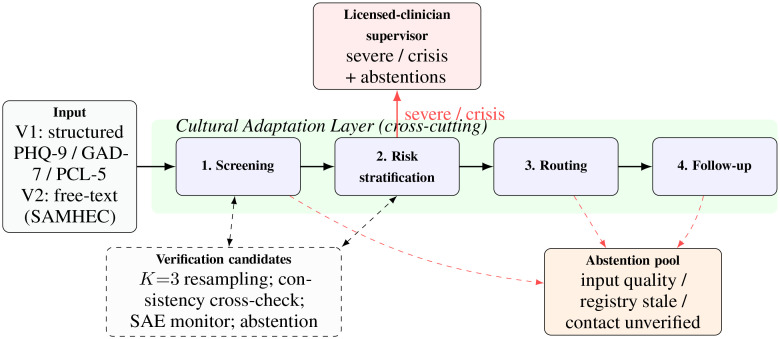
Proposed four-agent pipeline architecture as a clinical decision-support pathway. The Input block distinguishes Version 1 input (structured PHQ-9/GAD-7/Arabic PCL-5 administration) from Version 2 input (free-text Syrian Arabic, contingent on SAMHEC and Phase 1 validation). Solid black arrows mark consultative recommendations along the pipeline. Dashed red arrows from each of Agents 1, 3, and 4 mark explicit ABSTAIN paths to a separate abstention pool reviewed by the moderate-tier supervisor, with the abstention reason logged (input quality/clinical uncertainty/registry stale/contact unverified). The severe/crisis branch from Agent 2 escalates upward to the licensed-clinician supervisor and is a mandatory HITL checkpoint. The Verification Candidates panel (dashed-border box) sits below the pipeline and cross-checks the high-stakes stage; the Cultural Adaptation Layer is shown as a band intersecting every agent rather than as a *post-hoc* filter. Status: conceptual design specification; no component is implemented as a working prototype, and no end-to-end behaviour has been clinically evaluated.

The framework comprises four sequentially connected agents operating through a cross-cutting Cultural Adaptation Layer (CAL) under Human-in-the-Loop (HITL) governance. Each agent receives structured input from its predecessor and produces typed output for its successor. The complete pipeline architecture is presented in **Figure 2**, and formal agent specifications are provided in **Table 4**.

**Table 4:** Formal agent specifications: input/output schemas and core algorithms.

Agent	Input schema	Output schema	Core method	Escalation / abstention trigger
Screening	Text (Syrian Arabic); session ID; timestamp. Audio admitted only via CHW-mediated ASR transcription (Version 2), with ASR confidence propagated as an input-uncertainty term	Symptom vector s ∈ [0,1]d mapped to PCL-5, PHQ-9, GAD-7; confidence c; or ABSTAIN	Fine-tuned AraBERT/MAR- BERT; multi-label classification with K = 3 resampling for severe/crisis cases	c < 0.6 or SINdex-style inconsistency > threshold: ABSTAIN + flag for human review
Risk Stratification	Symptom vector s; confidence c; user history	Severity tier τ ∈ {mild, moderate, severe, crisis} or ABSTAIN; NL justification J with faithfulness flag	Threshold classification; SHAP (32) + faithfulness check (30)	τ ∈ {severe, crisis} or ABSTAIN: mandatory HITL
Routing	Severity τ; justification J; anonymised geolocation	Matched resource R from registry; rationale; wait time; or ABSTAIN	Rule-based mhGAP align- ment	No resource or registry stale: ABSTAIN + alert supervisor
Follow-up	Session ID; historical s vectors; previous τ	Updated τ′; trajectory; re-routing recommendation; or ABSTAIN	Longitudinal tracking at 1-wk, 1mo, 3-mo inte- rvals	Deterioration: push alert within 24h; missing data: ABSTAIN

Each agent may return an explicit ABSTAIN output alongside its typed payload (Section 2.3.6).

#### 2.3.1 Agent 1: screening Agent

The Screening Agent is designed for one of two interaction modalities: (a) a user-initiated text-based session, in which the help-seeker types in Syrian Arabic on a personal or shared device, and (b) a community-health-worker–mediated session, in which a trained CHW conducts a structured intake. Voice input is admitted only in the CHW-mediated case and only via an upstream automatic speech recognition (ASR) component; voice-only deployment without CHW mediation is explicitly out of scope for Version 1 (see Section 5.3) and is excluded from **Table 4**. Informed consent procedures, screen-privacy considerations, and shared-device scenarios are addressed in Sections 2.4 and 2.5.

When audio input is admitted (Version 2, CHW-mediated), speech recognition is an upstream dependency, not an in-scope component of this manuscript. Its known accuracy limits for Syrian Arabic are inherited as a constraint, and they propagate into the Screening Agent’s outputs in two ways that must be modelled, not ignored. (i) Word-level transcription errors (substitutions, deletions, insertions) introduce noise into the symptom-relevant lexical signal, which is most consequential for short utterances containing rare or dialectal terms (e.g., *ma’anat*, religious idioms) where a single misrecognised token can shift the inferred symptom domain. (ii) *ASR confidence drops* on noisy audio, accented speech, or whispered/quiet disclosures (which themselves correlate with stigma-laden content). The Screening Agent therefore accepts an ASR confidence score per utterance and propagates it into the overall input-uncertainty term carried by *c*: utterances with low ASR confidence are weighted down in the symptom-vector aggregation, and a session whose mean ASR confidence falls below a pre-specified floor triggers an explicit ABSTAIN with a flag indicating “input quality” rather than “clinical uncertainty”. The two abstention reasons are logged separately because they have different implications for the supervisor (one calls for re-recording, the other for clinical review). This handling describes design intent for Version 2 and is contingent on the prior availability of a Syrian-Arabic-capable ASR component whose error profile has itself been characterised; we do not assume that profile in the present manuscript.

The Screening Agent performs initial symptom detection from conversational Syrian Arabic input. It employs a fine-tuned transformer architecture (AraBERT-v2 or MARBERT) with a dialect-aware tokenizer that handles Syrian/Levantine Arabic, code-switching (Arabic–French–English), and dialectal orthographic variation. The agent extracts symptom-relevant signals through multi-label classification, producing a *d*-dimensional symptom vector **s** ∈ [0,1]*^d^* where each dimension corresponds to a validated clinical domain (PCL-5 intrusion, avoidance, negative cognition, arousal; PHQ-9 anhedonia, depressed mood, sleep, fatigue, appetite, guilt, concentration, psychomotor, suicidality; GAD-7 anxiety, worry control, restlessness, fatigue, concentration, irritability, muscle tension).

Importantly, the mapping from open conversational text to the symptom domains of PCL-5, PHQ9, and GAD-7 is not equivalent to structured administration of the instruments themselves. Validated scale thresholds (PCL-5 ≥ 33, PHQ-9 ≥ 15, GAD-7 ≥ 10) were established for the standardised, self-report or interviewer-administered forms with their original item phrasings, not for model-reconstructed probabilistic features. Treating the symptom vector s as a proxy for the corresponding scale score is therefore a methodological assumption that itself requires separate validation. Phase 1 of the validation roadmap addresses this by comparing free-text–derived symptom vectors against parallel structured-instrument administration on the same participants; the threshold-based stratification rules in Agent 2 are not considered clinically valid until that comparison is performed. For this reason, the first deployment version uses structured Arabic-language administration of validated translations of PHQ-9 and GAD-7 (Section 5.3); free-text symptom extraction is staged as a second-version capability contingent on this validation.

The conversational interface uses open prompts framed in culturally appropriate terms (“How have you been feeling lately?”/”What difficulties have you faced in daily life?”), avoiding clinical terminology. A confidence score *c* ∈ [0,1] accompanies each signal vector; outputs with confidence below the screening threshold (*c <* 0.6) trigger an explicit ABSTAIN return rather than a low-confidence prediction, and the case is flagged for human review. Abstention is exposed as an end-to-end pipeline output to ensure that downstream agents and human supervisors are not asked to act on unreliable signals.

#### 2.3.2 Agent 2: risk stratification agent

The Risk Stratification Agent classifies the symptom vector into four severity tiers: mild, moderate, severe, or crisis, or returns an explicit *abstention* (ABSTAIN) when evidence is insufficient to support a tier assignment with calibrated confidence above a tier-specific threshold. Treating abstention as a first-class output, rather than a side-effect of low confidence, follows recent recommendations for safety-critical scientific reasoning, where unsupported answers are more harmful than declining to answer (31). Tier assignment follows validated clinical thresholds adapted from instrument scoring: PCL-5 ≥ 33 (severe PTSD), PHQ-9 ≥ 15 (moderately severe depression), GAD-7 ≥ 10 (moderate anxiety), with crisis designation triggered by any indication of suicidal ideation, acute psychosis, or immediate safety risk. Any abstention is routed directly to a human supervisor with the underlying symptom evidence and confidence score.

The agent incorporates SHAP-based explainability (32) as a starting point for generating a natural language justification for each classification that identifies the symptom signals driving the severity assessment. We acknowledge that SHAP, when applied to deep transformer models, is not guaranteed to produce *faithful* explanations in the technical sense—attribution scores can diverge from the features that causally drive the model’s output (30). Phase 1 of the validation protocol therefore includes an explicit faithfulness check (Section 4.2), in which a held-out subset of explanations is evaluated for input-perturbation sensitivity and consistency with sparse-autoencoder–based feature attributions (30); explanations failing the faithfulness check are flagged in the audit log rather than presented to clinicians as if they were reliable causal accounts. The justification serves dual purposes: an audit trail for human supervisors and a structured clinical handover when the case is escalated.

The Risk Stratification Agent receives both a symptom vector and an upstream confidence score from the Screening Agent. We are explicit about three distinct quantities: (i) model confidence in symptom extraction (upstream, from Agent 1); (ii) tier-assignment confidence, computed locally by Agent 2 from the symptom vector and a calibrated decision rule; and (iii) the tier itself, which is a consultative output, not a clinical severity judgement. Tier-assignment confidence is computed as a monotone function of the minimum component-wise confidence in the relevant symptom subset, ensuring that uncertainty does not silently disappear across pipeline stages. When upstream confidence is low or symptom-vector components are themselves close to the decision boundary, the agent returns ABSTAIN rather than a tier. The output tier is consultative throughout: it is a recommendation for a routing destination, not a clinical severity diagnosis, and it does not authorise pharmacological or other clinical action.

#### 2.3.3 Agent 3: routing agent

The Routing Agent matches classified severity to available care resources via rule-based mapping aligned with mhGAP tiered care: mild cases receive psychoeducation chatbot and self-help resources; moderate cases connect to mhGAP-trained CHWs; severe cases are referred to the nearest psychiatrist; crisis cases trigger immediate human contact through pre-established local crisis protocols.

The agent queries a live resource registry—a RESTful API maintaining the current inventory of MHPSS services in the deployment region, with geolocation matching, availability status, and estimated wait times. This registry addresses the rapidly changing MHPSS service landscape in Northwest Syria. Every routing decision is logged with timestamp, inputs, matched resource, and rationale.

The Routing Agent’s correctness depends on assumptions about the surrounding care system that this article can describe but not yet satisfy. (i) Registry maintenance: the resource registry must be kept current by a partner organisation. We propose that the partner NGO (Section 2.4) holds primary maintenance responsibility, with daily synchronisation of availability and weekly verification of capacity, but we acknowledge that the WHO 4W mapping matrices on which initial registry contents are based are themselves updated quarterly at best. (ii) Specialist capacity: the specialist-to-population ratio in Northwest Syria (4) implies that even an optimally routed system will produce more severe-tier referrals than receiving psychiatrists can absorb. The Routing Agent must therefore distinguish “referred and reachable” from “referred and queued”; the latter is logged as an unmet need and surfaced to the supervisor digest, but does not itself trigger an alert unless wait time exceeds a pre-specified threshold. (iii) Crisis routing under specialist scarcity: for crisis cases, the routing target is the local crisis protocol of the partner NGO (which may include emergency department referral, CHW emergency response, or partner-coordinated transport), not a psychiatrist; this reduces dependence on individual specialist availability. (iv) Registry staleness: if the registry has not been synchronised within a pre-specified interval (default: 48 hours), the agent returns ABSTAIN for the routing step and the case is escalated to the supervisor for manual routing. These four assumptions are part of the conditional status of the framework and require operational partnership before they can be tested.

#### 2.3.4 Agent 4: follow-up and monitoring agent

The Follow-up Agent manages longitudinal engagement at defined intervals (1 week, 1 month, 3 months), re-administering the conversational screening protocol and comparing updated symptom vectors against the baseline trajectory. Deterioration is defined as an upward tier shift or emergence of crisis signals, triggering automatic supervisor alerts within a 24-hour response window.

Longitudinal re-contact in a conflict-affected population cannot assume stable communication channels, persistent device ownership, or continuous geographic stability. The agent design therefore embeds four specific accommodations. (i) Missing-response interpretation: a missed follow-up window is not automatically interpreted as deterioration or as engagement loss; it is logged as “contact unverified” and the agent retries through escalating channels (in-app, SMS, partner-NGO outreach) before returning ABSTAIN on the deterioration question. (ii) Phone loss and number change: at consent, the user is offered an optional secondary contact (a designated trusted person or a partner-NGO case manager); without a secondary contact, follow-up is best-effort only and the user is informed of this at consent. (iii) Shared-device scenarios: the session UI supports session-end “forget me on this device” actions and does not store conversational content locally; re-identification across sessions on the same device requires explicit re-authentication. (iv) User displacement: location data, where granted, is used only to update the resource registry mapping and is not retained as a stable user attribute. (v) Help-seeking visibility risk: follow-up messages are templated to be non-disclosing in their notification text. These accommodations describe design intent; their adequacy is an empirical question to be tested in Phase 3.

#### 2.3.5 Inter-agent communication protocol

Agents communicate via structured JSON messages over an internal message bus. Each message contains: (i) sender agent ID, (ii) receiver agent ID, (iii) timestamp, (iv) payload (typed according to the output schema in **Table 4**), and (v) a cryptographic hash for audit integrity. Pipeline execution is sequential with mandatory HITL checkpoints at the Risk Stratification and Routing stages. Agent failures (timeout, low confidence, exception, or explicit abstention) trigger graceful degradation: the pipeline halts and escalates to the human supervisor rather than proceeding with unreliable output.

#### 2.3.6 Verification, hallucination mitigation, and abstention

A strictly feed-forward four-agent pipeline—in which each agent’s output is consumed by the next without cross-checking—would be insufficient for any system claiming a 95% crisis-sensitivity floor in this setting, given documented evidence that LLM and transformer outputs can be fluent yet logically or factually unsound (29, 30). Recent multi-agent reasoning work also shows that introducing parallel inference and cross-checking, rather than a single linear chain, materially improves reliability and interpretability over Chain-of-Thought–style decoding (21).

We propose three candidate verification mechanisms as experimental hypotheses for future evaluation, not as a ready-made safety layer. The methods described below were developed and evaluated on English language general-purpose reasoning and question-answering benchmarks, not on Syrian Arabic clinical conversational input. Their transferability to the present context is itself an open empirical question and is included in Phase 1 of the validation roadmap. We adopt these candidates because the alternative—a strictly feed-forward pipeline at a high crisis-sensitivity floor—is worse, not because the candidates are demonstrated to be sufficient. The Phase 1 evaluation is designed to falsify any of them: a mechanism that fails to improve abstention-coverage or risk-at-coverage on Syrian Arabic clinical input relative to a no-verification baseline is dropped, not retained.

Candidate 1: Semantic consistency cross-check. For severe and crisis tier assignments, the Screening Agent’s symptom vector would be regenerated via *K* = 3 resampled passes over the same input. A semantic inconsistency index in the spirit of SINdex (29) would be computed over the resulting signal sets via hierarchical clustering of sentence-embedded symptom narratives; cases with inconsistency above a calibrated threshold would be routed to abstention even if individual-pass confidence is high. The mechanism would convert the pipeline into a parallel-then-aggregate structure at the highest-stakes stage. It remains an open question whether the inconsistency index, defined for English-language reasoning benchmarks, behaves as a useful signal on Syrian Arabic clinical conversational input.

Candidate 2: Sparse-autoencoder feature monitor for user-facing output. Any free-form text produced for the user (psychoeducation messages, follow-up prompts, supervisor handover summaries) would be screened by a sparse-autoencoder–based feature monitor in the spirit of SAFE (30), configured to suppress outputs whose internal representations align with known fabrication features. Outputs failing the screen would not be emitted; the pipeline would instead return a templated, evidence-grounded fallback drawn from validated mhGAP (6) content. It is likewise unverified whether the feature monitor’s discriminative power transfers to a clinical-Arabic distribution it was not trained on.

Candidate 3: Abstention as a first-class output. Each agent in **Table 4** can return ABSTAIN alongside its typed payload. Abstention is not a silent failure mode: it is explicitly logged, surfaced to the human supervisor, and counted as its own outcome category in evaluation. Following (31), we consider abstention coverage and risk-at-coverage metrics alongside conventional accuracy in the validation roadmap, on the principle that an unsupported clinical conclusion in a fragile-state setting is more harmful than declining to conclude. What coverage level is acceptable to supervising clinicians, given the workload it generates, is a further open question.

These three candidates are proposed for Phase 1 evaluation alongside the components they augment. They are not substituted for the human-in-the-loop checkpoints described in Section 2.5: the safety floor of the system is the human supervisor at the severe/crisis threshold, with or without the verification candidates. Any candidate that fails to improve abstention-coverage or risk-at-coverage on Syrian Arabic clinical input relative to a no-verification baseline is dropped from the architecture before subsequent phases begin.

### 2.4 Cultural Adaptation Layer

**Figure 3:**
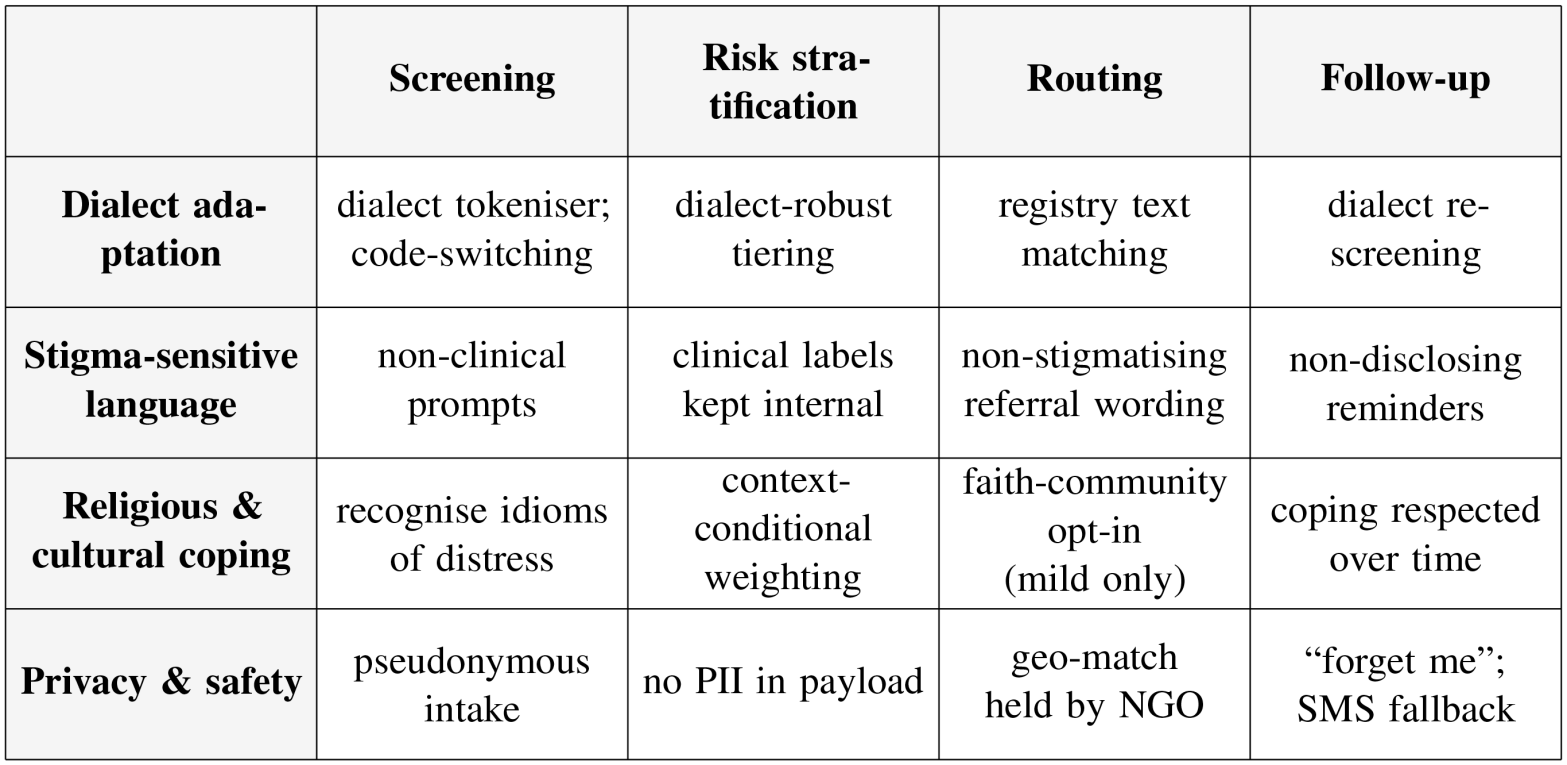
Cultural Adaptation Layer operationalisation matrix: four cross-cutting design dimensions (rows) by four pipeline agents (columns). Each cell specifies the operational contribution of the row’s CAL dimension at the column’s pipeline stage; none of the cells is empty, which is the analytical content of “cross-cutting” — the CAL is not a *post-hoc* filter applied after the pipeline output but is operative at every agent. Source-literature anchoring for each row is recorded in **Table 3**: Arabic clinical NLP for the dialect row (12, 13); cross-cultural psychiatry for stigma-sensitive language and religious coping (28); LLM reliability literature for privacy-aware sanitisation (30). Status: conceptual design specification; cultural validity would be tested empirically in Phases 2 and 3 of the validation roadmap.

The Cultural Adaptation Layer (CAL) is a cross-cutting design constraint applied across all four agents. It operationalises the Cultural Gap through four components, as shown in **Figure 3**.

Dialect adaptation. All agent interfaces operate in Syrian/Levantine Arabic, not Modern Standard Arabic (MSA). This requires dialect-aware tokenization and normalization procedures mapping dialectal variants to MSA representations for downstream NLP processing. The system accommodates code-switching common in urban Syrian and refugee speech (Arabic–English, Arabic–French, and—in displacement settings—Arabic–Turkish). Code-switching behaviour is treated as an empirical question to be tested rather than a property to be assumed: Phase 1 of the validation protocol includes a dedicated code-switching evaluation track (Section 4.2) drawing on recent multilingual NLI evaluation methodology (21) and adapted for the Syrian Arabic clinical setting. Recent evidence that judge-side language localisation can invert backbone rankings in agentic evaluation (33) also motivates running each evaluation in both Arabic and English judge configurations, to ensure that performance estimates are not artefacts of the evaluation language.

Stigma-sensitive language. All user-facing output replaces clinical terminology with culturally appropriate formulations: *ma’anat* (suffering), “difficulties after what you have been through,” “the weight you are carrying.” The term *junun* is never generated. Internal system logs retain clinical labels for supervisor use.

Religious and cultural coping. Religious coping expressions—faith, prayer, acceptance of divine will—are not treated as automatic positive resilience signals. The cross-cultural psychiatry literature on Syrian conflict populations (28) establishes that religious coping is frequently adaptive in this setting, but the same literature also notes that religious framings can serve avoidance functions, can mask depressive hopelessness as fatalistic acceptance, and can be invoked under social pressure to disclaim distress. The CAL therefore treats religious coping language as a context-conditional signal rather than as a uniformly positive marker: in the absence of accompanying severity indicators (e.g., active suicidal ideation, severe functional impairment, somatic symptom load above tier threshold), religious coping language is flagged as a candidate resilience signal that the human supervisor may weigh; in the presence of severity indicators above the moderate tier, religious coping language is not used to down-weight the severity assessment, and the case is escalated on the severity indicators alone. The classification rule is conservative on the down-weighting side by design: under-weighting an apparent resilience signal is clinically less consequential than under-tiering a severe case.

Faith-community referral as an opt-in mild-tier destination. The Routing Agent’s registry may include faith-community leaders as referral destinations only for mild-tier cases, only where the user has explicitly opted in to such routing at consent, and only where the partner NGO has independently vetted the named faith-community contact for safeguarding standards. The ethical justification is conditional rather than universal: in Syrian populations and the diaspora, community-based help-seeking through faith networks is a documented and frequently preferred pathway for distress that does not meet a clinical threshold, and excluding it would force a Western secular care model onto users who may legitimately prefer a faith-community first contact. The risks are equally real: routing to a faith-community contact a user did not opt into would breach autonomy; routing to a contact whose safeguarding posture is unknown would create harm potential; and routing severe-tier cases to faith communities rather than to clinical care would substitute spiritual support for clinical treatment in a context where the latter is required. The three conditions above (mild-tier only, explicit opt-in, NGO vetting) are intended to permit the route where it is appropriate and to foreclose it where it is not. We acknowledge that the operational adequacy of this allocation cannot be assumed and must be tested in Phase 3 qualitative evaluation.

Privacy and safety norms. The system separates *pseudonymisation* (used at runtime, with linkage keys held under controlled access by a partner NGO) from *anonymisation* (irreversible, used for any dataset released for research or external audit). The distinction matters in this context: longitudinal follow-up at 1-week, 1-month, and 3-month intervals requires re-identification of the same individual across sessions, which is incompatible with full anonymisation. Linkage keys are held by the partner NGO, never co-located with the AI system or its operators, and are protected by separation-of-duties access controls. Session payloads transmitted between agents and to the supervisor dashboard contain pseudonymous IDs only; no personally identifiable information is stored on the system. All transmissions are encrypted in transit and at rest. The architecture explicitly accounts for surveillance risks specific to conflict-affected populations and the social risk of visible mental health help-seeking, including support for shared-device scenarios and an option for anonymous one-shot screening (without longitudinal linkage) where the user declines follow-up consent.

Data life cycle and role separation. The data life cycle requires three roles to be separated, both for confidentiality and for accountability. (i) The partner NGO (deploying entity in the region) controls linkage keys, holds clinical responsibility through its supervising clinicians, controls the on-the-ground informed-consent process, and decides what data leaves the deployment region; it is also the controller of record under applicable data-protection law. (ii) The research group (the team developing the architecture and validation roadmap, including the present authors) accesses only anonymised data, by destruction of the linkage key, under a data-use agreement specifying purpose, retention, and re-share constraints; it does not access pseudonymous data and cannot re-identify any individual record. (iii) The technical operator (the entity hosting the model inference and the message bus) accesses pseudonymous session payloads at runtime for the purposes of inference and audit logging only, under a processing agreement with the partner NGO; it does not hold the linkage key and cannot re-identify records. The flow is therefore: identifiable data → partner NGO (controller) → pseudonymised at session start → technical operator (processor) → session output and audit log returned to partner NGO → at end of follow-up window, linkage key destroyed by partner NGO → anonymised dataset optionally released under DUA to research group. Geolocation matching, which carries re-identification risk in fragile settings, is performed by the partner NGO at registry-query time and is not retained downstream as a stable user attribute. None of this is operative without a written agreement covering all three roles; the absence of such an agreement at present is part of the conditional status of the framework.

### 2.5 Human-in-the-Loop governance

The HITL architecture establishes four governance mechanisms: (1) Threshold Escalation: mandatory human review for all severe/crisis classifications, with structured clinical summaries; (2) Routing Oversight: daily supervisor digest of routing decisions with override capability; (3) Deterioration Alerts: push notifications to supervisors within 24 hours of detected deterioration; (4) Audit Logging: complete decision trail for every case, accessible to supervisors and supporting post-incident review.

Responsibility model. The category of “supervisor” covers two distinct clinical roles, with distinct decision authority and distinct liability allocation. This distinction is not a nuance; it is the substantive core of the responsibility model.

- Decision authority for severe and crisis tier cases: the severe/crisis-tier supervisor (an mhGAP-trained licensed clinician, as defined in Section 1.1) holds sole decision authority. The system’s tier output and verification flags are advisory inputs to this clinician’s judgement; the clinician’s recorded decision is the operative one. The clinician may concur with the system, override it, request additional information, or refer outside the system. No severe-tier or crisis-tier action is taken on the basis of the system output alone.
- Decision authority for moderate tier cases: the moderate-tier supervisor (an mhGAP-trained CHW) holds decision authority for routing to a CHW counselling session or to standing self-help resources, under the standing protocol of the partner organisation. Cases at the moderate–severe boundary, or any moderate-tier case the CHW flags as ambiguous, are escalated to the severe/crisis-tier supervisor.
- Mild tier cases: routing is enacted automatically (psychoeducation, self-help resources). The moderate tier supervisor reviews mild-tier outputs in a daily digest with override authority but without a per-case pre-emission gate.
- Liability for erroneous system recommendation: clinical liability rests with the human decision-maker at each tier (the licensed clinician for severe/crisis; the CHW under the standing protocol of the partner organisation for moderate; the partner organisation itself for the standing mild-tier protocol). This is the standard allocation for clinical decision-support and not a novel claim; the system is positioned as a recommender, and the recommender does not carry clinical liability for the act-on or override decision. Where the operative question is whether the system’s recommendation was itself defective (for example, a missed crisis because of an upstream model failure), liability sits with the partner organisation as the deploying entity, under whatever formal indemnification arrangement is in place with the technical operator. This allocation is described in detail in the deployment-realism considerations of Section 5; we flag here that no allocation is operative until a deployment contract specifies it, and we therefore treat the responsibility model as part of the conditional status of the framework.
- Discrepancy resolution: where supervisor judgement diverges from the AI tier, the supervisor’s judgement is operative. Divergences are logged, summarised weekly, and used as training signal for model revision; they are not used to alter the supervisor’s standing.
- Expected supervisor workload: based on Phase 2 simulation estimates and partner-NGO intake volumes, we anticipate approximately 10–15 severe/crisis escalations per severe/crisis-tier supervisor per day at steady state. Abstention outputs are routed to the same queue with second priority. If the queue exceeds a pre-specified depth (default: 20 awaiting items), the Routing Agent down-rates its acceptance threshold and the system surfaces a capacity warning to the partner NGO. These workload estimates are expert assumptions to be revisited against partner-NGO data; they are not load-tested.
- Data life cycle: pseudonymous session payloads are retained for the maximum of 30 days or until the longitudinal follow-up window closes, whichever is later. Linkage keys (held by the partner NGO; see Section 2.4) are destroyed at the end of the longitudinal window, irreversibly anonymising the record. Users may request earlier deletion of linkage keys, which converts their record to anonymous form.
- Incident response: a single point of contact within the partner NGO is designated as the incident response lead. Incidents (missed crisis, harmful output, privacy breach, severe model failure) trigger immediate suspension of the affected agent pending review by a pre-constituted incident review panel (clinical lead, technical lead, ethics representative).

The system is explicitly positioned as augmentative: it multiplies CHW reach by pre-screening and triaging, concentrating limited specialist capacity on severe presentations. We are also explicit that the architecture, when considered with its HITL layer, is closer in genre to a hybrid clinical decision-support system than to an autonomous multi-agent pipeline: every consequential output is subject to human acceptance, modification, or override at the severe/crisis threshold.

### 2.6 Ethical guardrails

**Figure 4:**
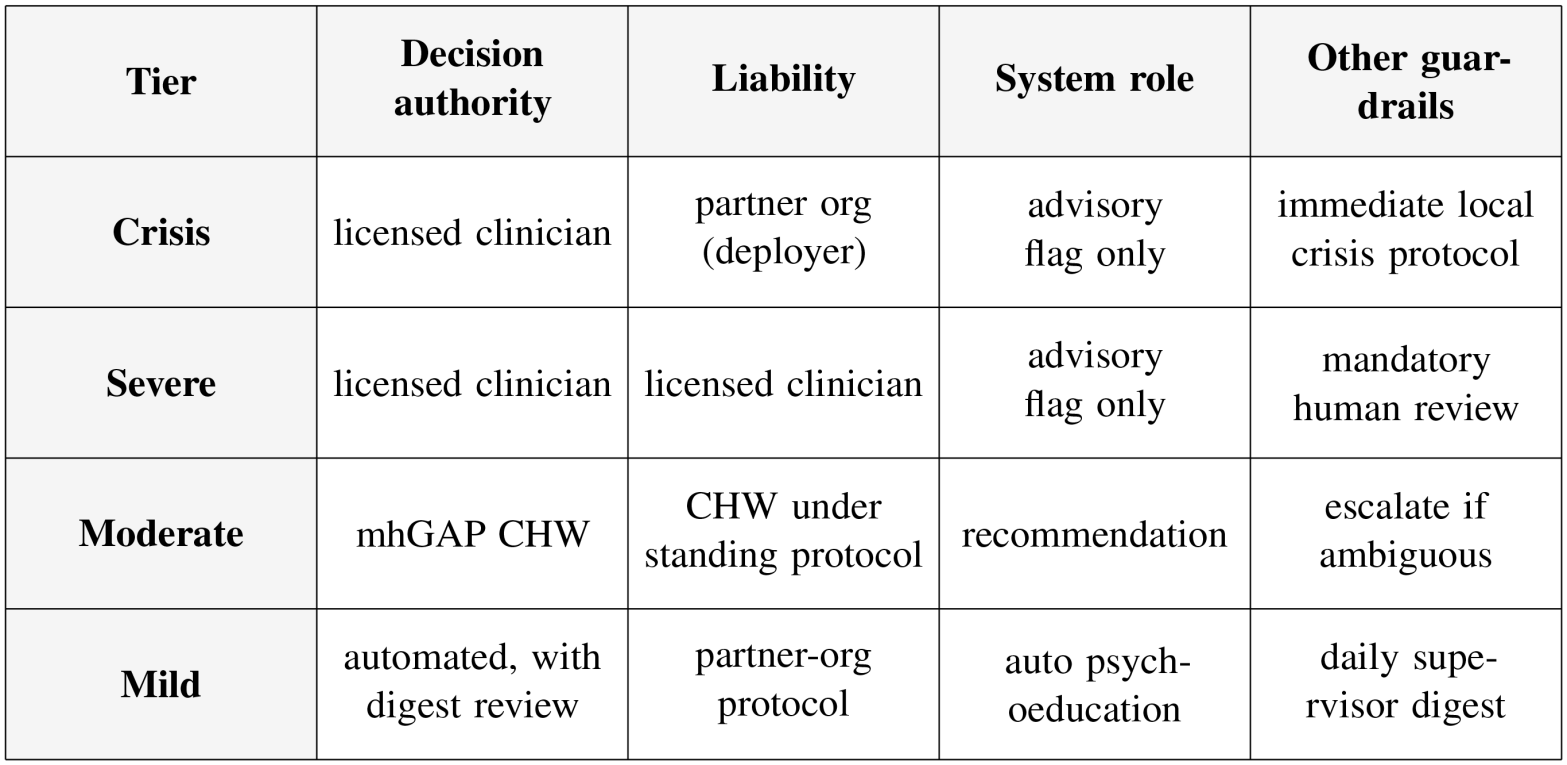
Decision-authority and liability allocation across triage tiers — the two-role responsibility model in tabular form. Rows correspond to the four severity tiers (crisis, severe, moderate, mild). Columns specify, at each tier: (i) which supervisor role holds decision authority, (ii) which party carries clinical and defective-recommendation liability, (iii) what the system contributes as advisory input, and (iv) the other applicable guardrails (privacy, equity, bias, audit). The figure operationalises the responsibility model of §2.5 as a tier-conditional allocation rather than as four parallel “ethical principles”. Across all tiers the system is positioned as a recommender, not a decision-maker; the boundary of clinical responsibility moves with the tier of the case, and privacy, equity, bias-audit, and audit-logging guardrails apply throughout. Status: design specification; operational adequacy in deployment has not yet been demonstrated and depends on partner-organisation consolidation.

The framework addresses four priority ethical dimensions for fragile-state AI deployment, illustrated in **Figure 4**.

Data privacy. Beyond conventional protections, the framework prohibits persistent storage of data identifying individuals as mental health help-seekers. Design accounts for surveillance infrastructure and ongoing informed consent appropriate to limited digital literacy.

Algorithmic bias. Arabic NLP models overrepresent MSA and Gulf/Egyptian dialects whilst underrepresenting Syrian/Levantine patterns. The framework mandates regular bias audits using representative Syrian Arabic test sets and diverse annotation teams.

Accountability. The system makes no clinical diagnoses; all routing decisions are advisory and subject to human override. Liability rests with the accepting human supervisor. Audit logs support post-incident review.

Equity of access. Any implementation must include low-bandwidth and SMS-fallback capability, address shared-device scenarios, and monitor whether uptake patterns reflect or exacerbate existing inequities.

## 3 Preliminary comparative mapping

### 3.1 Method and scope of the mapping

The interpretive procedure used to construct the comparison below is described in Section 2.1. We reiterate that the mapping is structured but not systematic, that no PRISMA registration was undertaken, and that the comparison is asymmetric—the proposed work is described from design intent whilst compared systems are described from published reports. It is therefore offered as a preliminary positioning exercise, and not as a demonstration of comparative superiority, evidentiary equivalence, or a substitute for systematic review.

### 3.2 Comparative mapping

The mapping is presented in two parts to avoid conflating the conceptual design intentions of the proposed framework with the evidentiary maturity of compared systems. **Table 1** records the design requirements of the proposed architecture — what a future Syrian-Arabic post-conflict triage system would need to satisfy — and notes, for each requirement, whether comparable mental-health frameworks address it. **Table 2** records the evidentiary maturity of the five compared mental-health frameworks against the same architectural dimensions, without recording entries for the proposed system (because, having no prototype, its only entry would be “conceptual” across the board). EMPATHIA, as a humanitarian allocation analogue rather than a clinical triage system, is presented in a separate panel (**Table 5**) with a narrative that makes the structural-comparator status explicit.

**Table 5:** EMPATHIA (20) as a humanitarian multi-agent analogue, presented separately because its task (refugee placement allocation) differs structurally from clinical triage.

Dimension	Proposed work	EMPATHIA (20)
Task type	Clinical triage (assessment → routing → follow-up)	Resource allocation (refugee placement selection)
Population	Syrian Arabic-speaking, post-conflict	Refugees, primarily Kakuma camp
Multi-agent decomposition	Four agents along mhGAP intake-to-disposition phases	Three agents (emotional, cultural, ethical) in a selector– validator structure
Cultural perspective	Cross-cutting Cultural Adaptation Layer applied at every agent	Dedicated cultural agent operating alongside emotional and ethical agents
Human role	Hybrid clinical decision-support; severe/crisis decisions taken by licensed clinician	Augmentative recommender; placement decisions taken by allocation authority
Verification	Three candidate verification mechanisms (consistency, SAE style monitor, abstention)	Selector–validator structure with cross-check between agents
Evidentiary status	Conceptual only; no prototype	Implemented and evaluated on 6,359-case Kakuma dataset
What is shared	Multi-perspective deliberation in a fragile-state context; augmenting rather than replacing human practitioners; cultural validity as a design constraint rather than a translation step	
What is not shared	Clinical triage logic and mhGAP alignment; clinical instrument scoring; longitudinal symptom-trajectory follow-up; clinical safety floor (95% crisis sensitivity)	

The shared and divergent dimensions are listed; clinical-psychiatric-triage dimensions are not coded because they are not in EMPATHIA’s scope.

Each row of **Tables 1** and **2** is coded against an explicit criterion, listed in the second column of **Table 1**. Coding rules: ✓^C^ = the cited work explicitly describes the requirement as design intent, with a textual statement to that effect; ✓^I^ = the cited work documents a working prototype that includes the corresponding capability, with sufficient methodological detail for the implementation to be identified; ✓^V^ = the cited work reports an independent evaluation of the capability against a held-out benchmark or human reference standard; — = the cited work does not address the requirement although it is within the scope of the work; N/S = the work is designed for a different problem and the requirement would not be expected. For “This Work”, all entries are ✓^C^ because no prototype, corpus, or empirical evaluation has yet been produced.

Why EMPATHIA is in a separate panel. EMPATHIA (20) is the closest published multi-agent system for a fragile-state population, but it is not a clinical psychiatric triage system: its task is refugee allocation amongst placement options, not assessment-to-care routing of mental-health help-seekers. Placing it in the same row-by-row comparison as the five mental-health frameworks above would force most of its cells into N/S and obscure what it actually shares with the present proposal. We therefore present it as a separate panel (**Table 5**) and discuss the substantive comparison narratively.

Five candidate design requirements emerge from this preliminary mapping—each currently a conceptual proposal in the present manuscript, and an empirically tested capability in some or all of the compared mental-health frameworks: (1) the Cultural Adaptation Layer combined with Arabic dialect support, which no compared mental health framework implements; (2) explicit triage and routing to a live resource registry, extending beyond assessment into care delivery; (3) longitudinal follow-up as a system component; (4) explicit verification, hallucination handling, and abstention mechanisms (Section 2.3.6); and (5) privacy architecture designed for surveillance-exposed, fragile-state contexts. We use the phrase candidate design requirements rather than differentiators to mark that these are properties a future Syrian-Arabic post-conflict triage system would need, not properties this manuscript has demonstrated. Each requirement is also a risk area: implementation depends on the missing SAMHEC corpus, local cultural validation, partnerships with MHPSS organisations, and supervisor capacity that have not yet been secured.

## 4 Validation roadmap (future work)

This roadmap is not an implementation plan or a pre-registered protocol. Its purpose is to identify the sequence of evidentiary conditions that would have to be met before progressively more demanding forms of evaluation could responsibly be attempted, and to be explicit that several of those conditions — a Syrian Arabic corpus, institutional partnerships, governance agreements, and independent ethical oversight — do not yet exist and may prove infeasible. It is a conditional sequencing argument, not evidence of operational readiness.

**Figure 5:**
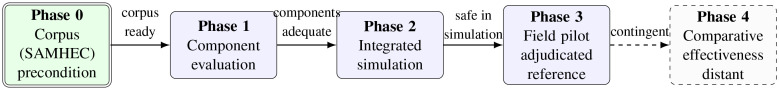
The validation roadmap as a sequencing argument. The five stages are shown with their categories made visually distinct: Phase 0 (double rule) is a corpus-construction precondition without which nothing else is meaningful; Phases 1–3 are evaluation stages of increasing realism, separated by explicit gates; and Phase 4 (dashed) is a distant comparative-effectiveness scenario, reached only through a conditional gate. The reference standard in Phase 3 is a clinician-adjudicated sub-sample rather than the community health worker, and the crisis-sensitivity figure obtained there is a lower bound because the most acute cases are excluded by design. Status: planning logic for future empirical work; numerical parameters are planning assumptions, not pre-specified targets; no phase has begun and no partnership agreements are signed.

The argument has five stages, shown in **Figure 5** and summarised in **Table 6**: a corpus-construction precondition (Phase 0); three evaluation stages of increasing realism (Phases 1–3); and a distant comparative effectiveness scenario (Phase 4), whose feasibility depends on the outcomes of the earlier stages and on a stable deployment context that does not presently exist. Phase 0 is a genuine precondition rather than a scheduling detail: until a Syrian Arabic corpus exists there is no baseline against which the conversational components can be evaluated, no basis for re-deriving tier thresholds, and no reference for monitoring change over time. Phase 4 is described for completeness of the argument, not as a planned activity. The few numerical values that appear in what follows — indicative corpus and sample sizes, an agreement coefficient, a crisis-sensitivity floor — are planning assumptions included only to fix orders of magnitude; they are not targets derived from existing data, and the ordering of the stages matters more than any of the figures.

**Table 6:** The validation roadmap as a five-stage sequencing argument.

#	Stage	What it would aim to establish	Precondition
0	SAMHEC corpus construction	A Syrian Arabic, clinically annotated reference corpus, without which no conversational component can be evaluated	Partnership agreements and ethical approval
1	Component-level evaluation	Whether each component performs adequately on Syrian Arabic input, including the free-text-versus-structured comparison that distinguishes the two versions	Phase 0 complete
2	Integrated simulation	Whether the assembled pipeline behaves safely and coherently on realistic simulated cases	Phase 1 complete
3	Controlled field pilot	Whether triage recommendations agree with a clinician-adjudicated reference under real workflow conditions, and whether the system is culturally acceptable	Phase 2 complete
4	Comparative effectiveness (distant)	Whether AI-augmented triage improves appropriate-care rates relative to standard task-shifting	Contingent on Phases 0–3 and a stable deployment context

Phase 0 is a precondition; Phases 1–3 are evaluation stages of increasing realism; Phase 4 is a distant scenario described for completeness, not a planned activity. The table states what each stage would aim to establish and what must precede it, rather than fixed performance targets.

### 4.1 Phase 0: SAMHEC corpus construction (precondition)

The Syrian Arabic Mental Health Evaluation Corpus (SAMHEC) is the rate-limiting resource for the entire validation programme. We place it as a distinct Phase 0 rather than as part of Phase 1 because nothing else in the roadmap is meaningful without it: without SAMHEC there is no Syrian Arabic baseline against which the Screening Agent can be evaluated, no source for tier-threshold re-derivation, and no reference benchmark for drift monitoring. The corpus is therefore not an auxiliary deliverable of Phase 1; it is the precondition that makes Phase 1 a coherent activity at all.

The corpus would be built from de-identified conversational transcripts of existing MHPSS intake sessions, obtained through partnership with operating NGOs under ethical approval, and annotated by clinical psychologists together with native Syrian Arabic speakers for symptom-relevant signals mapped to the PCL-5, PHQ-9, and GAD-7 domains, with disagreements adjudicated by a senior clinician and inter-annotator agreement assessed before the corpus is treated as usable. Its scale should be adequate to support component evaluation rather than fixed in advance, and its construction is gated on formal data-sharing agreements that keep linkage keys with the partner NGO; no such agreements are yet in place, and Phase 0 cannot begin until they and the corresponding ethical approvals exist. Phase 1 does not begin until the corpus is adequate in size and annotator agreement, passes a representativeness audit against partner-NGO catchment demographics (age, gender, displacement status, dialect variation), and has the ethical approvals required for the later phases; failure on any of these returns the programme to corpus work rather than advancing it.

### 4.2 Phase 1: component-level technical benchmarking

Phase 1 evaluates each component on the Syrian Arabic corpus and on synthetic material derived from it, and cannot begin until Phase 0 is complete. The central test for the Screening Agent is a comparison of its free-text symptom output against parallel structured-instrument administration on the same participants: this comparison is the gate between the two versions of the system (Section 5.3), since until free-text extraction reaches calibrated equivalence with structured administration the free-text version is not authorised for the later phases. Two further questions bear on the linguistic constraint: how performance on code-switched input (Arabic–English, Arabic–French, and, in refugee-hosting communities, Arabic–Turkish) compares with monolingual baselines, evaluated under both Arabic and English judge configurations to guard against the language-as-confound problem documented for agentic evaluation (21, 33); and whether dialect robustness can be held to a stricter standard at the severe and crisis tiers, where false negatives are least acceptable, than at the milder tiers.

Component evaluation also covers symptom-extraction quality, calibration, and abstention behaviour, treating selective answering as itself a clinically meaningful capability (31); and, for the Risk Stratification Agent, an explicit check of whether its SHAP-based explanations (32) are faithful to the features actually driving the model, compared against input-perturbation sensitivity and sparse-autoencoder attributions (30), with explanations that fail the check flagged in the audit log rather than shown to clinicians as reliable. Screening baselines would include fine-tuned AraBERT-v2, MARBERT, and CAMeL-BERT against zero-shot GPT-4o with Arabic prompting, following Fouda et al. (13), and the Routing and Follow-up agents would be exercised on simulated registries and longitudinal trajectories for appropriate routing, graceful degradation when resources are unavailable, and timely deterioration detection.

### 4.3 Phase 2: integrated system simulation

Phase 2 would assemble the components into the full pipeline and exercise it on a body of high-fidelity clinical simulation scenarios co-designed with MHPSS practitioners and stratified across severity tiers, presenting conditions, degrees of cultural complexity (somatic idiom-dominant, religiously-framed, and stigma-avoidant presentations), and edge cases such as suicidal ideation requiring immediate escalation. The questions at this stage are whether the assembled system routes cases coherently from end to end; whether it ever misses a crisis escalation, the safety property that must hold before any contact with real help-seekers; whether its user-facing language remains stigma-sensitive and free of clinical jargon, as judged by bilingual clinical reviewers; and whether human escalation and audit logging behave as specified. Adequacy at this stage is a precondition for the field evaluation that follows, not a substitute for it.

### 4.4 Phase 3: controlled field pilot

Phase 3 would be a mixed-methods pilot in partnership with MHPSS organisations in Northwest Syria or in Syrian refugee-hosting communities, under local ethical approval and with informed consent obtained in Syrian Arabic, including verbal explanation to accommodate limited literacy. Adult help-seekers presenting to participating services would be assessed in parallel by the system and by a trained mhGAP community health worker, with the system’s recommendations recorded but not acted upon; routing would continue to follow the worker’s judgement. Crucially, the community health worker’s assessment is not treated as the reference standard: a purposive sub-sample, with full coverage of the severe and crisis tiers, would be independently adjudicated by a licensed clinician blinded to both the system and the worker, and that adjudicated assessment would be the reference for the agreement analysis. The pilot’s exclusion of people in immediate danger means that any crisis-sensitivity figure it yields is a lower bound on a less acute portion of the crisis spectrum, not an estimate for the full deployment population, and we would report it with that limitation rather than generalise from it. Alongside the quantitative comparison of triage agreement, the pilot would include a qualitative strand—interviews with help-seekers and focus groups with community health workers—on cultural appropriateness, comfort, trust, and workload, analysed against the Triple Gap dimensions; it would run under an independent safety-monitoring arrangement empowered to halt it in the event of missed crises or reported harm, and under the privacy regime described in Section 2.4.

### 4.5 Phase 4: comparative effectiveness evaluation (distant scenario)

Phase 4 is described only to complete the sequencing argument. The conventional design for evaluating a care-pathway change of this kind is a stepped-wedge cluster-randomised trial (34), in which sites move sequentially from standard mhGAP triage to AI-augmented triage with community-health-worker oversight; its primary outcome would be the proportion of help-seekers receiving severity-appropriate care within a short window of first contact, with secondary attention to worker throughput and burden [for example, using a validated measure such as the ProQOL (35)], time-to-care, retention, and earlier deterioration detection, analysed with mixed models that account for site-level clustering. Such a trial is a distant prospect, contingent on a successful exit from Phase 3, on stable infrastructure across several sites, on a partner funding and security horizon compatible with a multi-month trial, and on regulatory approval at each site. None of these conditions is currently satisfied; the sample size, the cluster structure, and the effect size that such a trial would target are questions for the point at which it becomes feasible, not commitments made here.

### 4.6 Cross-cutting validation considerations

Several considerations cut across the stages. Each evaluation would be disaggregated by gender, age, displacement status, and dialect region, with disparities beyond a pre-agreed margin treated as a reason to revise the model before proceeding, and the integrated-simulation stage would include adversarial inputs designed to probe behaviour under ambiguity and manipulation. Because the later stages would unfold in an unstable setting—shifting demographics, evolving conflict, and gradual change in dialect and idiom—the roadmap treats distributional drift as a first-order concern rather than a *post-hoc* one, monitoring population, input, and performance drift against frozen baselines. It also fixes in advance the rule that a model is not silently retrained in the middle of a comparative trial: the system under evaluation must remain a stable object, so retraining during a trial is permitted only for a documented safety incident and is then treated as the start of a new evaluation rather than a continuation. Finally, evaluation code, synthetic-data generators, scoring rubrics, and anonymised results would be deposited openly, following FAIR principles (36), with simulation templates shared to support independent replication.

## 5 Discussion

This article has presented a conditional conceptual hypothesis, not a demonstrated system. Its contribution, in so far as it has one, is integrative: it brings together in a single argument three commitments that the multi-agent mental-health frameworks we surveyed do not jointly make — the integration of multi-agent triage with mhGAP task-shifting and cultural adaptation, adaptation to the specific constraints of fragile state deployment, and a staged argument for how the proposal could be tested. None of these is established here; each is a design intention whose adequacy could be assessed only once a corpus exists, partnerships are in place, and the staged evaluation of Section 4 has been carried out. We discuss, in that light, the limitations that bear most directly on whether the proposal could ever be realised, and then return to what the two configurations of the system would, and would not, put to the test.

### 5.1 Deployment realism

Existing literature on AI in healthcare has repeatedly documented a gap between technical potential and real-world clinical impact: AI is over-promised and under-delivered, and adoption depends on systematic introduction into care pathways rather than on algorithm performance alone (26). A survey of AI and decision support systems in psychiatry found small training samples, an emphasis on algorithm development without real-world interaction, and methodological shortcomings in evaluation, leading the authors to conclude that decision support in psychiatry is not yet ready for routine deployment (25). We take these findings seriously and acknowledge several deployment-realism limitations specific to the Syrian context.

The most consequential limitation concerns data acquisition: the validation roadmap is gated on SAMHEC, which depends on partnerships with MHPSS organisations operating under conditions of insecurity, NGO funding-cycle volatility, and population mobility. Ethical risks are non-trivial: meaningful informed consent for clinical-data reuse is harder to secure in displacement settings, where populations are exposed to surveillance risks and may have limited capacity to refuse a request without reputational or service-access cost. Logistically, secure data transfer infrastructure may be intermittent, and partner organisations may lack legal capacity to enter formal data-sharing agreements with academic institutions outside the country. We have flagged SAMHEC partnership consolidation as a precondition for Phase 1 and have not assumed it.

Second, conflict zones produce rapidly changing populations: new displacement events alter demographic composition, prolonged exposure shifts the symptom profile, and language and idiom shift over time as host-community contact varies. Drift monitoring (see Section 4, Cross-cutting validation considerations) is necessary but not sufficient; it can detect drift but cannot guarantee that retraining data will be available when drift is detected, particularly if a partner NGO loses access to a region. We acknowledge this as a residual risk and treat it as a reason to favour modular, locally retrainable model components over monolithic large-model dependencies.

Third, the framework depends on a fragile chain: NGO partners with security access; a registry of MHPSS resources that must be kept current; supervisor capacity to handle escalations; encrypted infrastructure with reliable power and connectivity. Each link is precarious in Northwest Syria and similar settings. A realistic deployment plan must include redundancy (multi-NGO partnerships rather than single dependency), low bandwidth and SMS fallback, and explicit decommissioning protocols for the case where an NGO partner withdraws or a region becomes inaccessible.

Finally, stigma around mental illness is high in Syrian populations, and visible help-seeking carries social risk (28). AI specifically introduces additional concerns: surveillance fears (warranted, given the conflict context), distrust of digital systems, and the well-documented preference for human-derived clinical decision support over AI-derived support in depression care reported in the broader literature. The system’s design accommodates these concerns (pseudonymous one-shot use, stigma-sensitive language, opt-in follow-up), but acceptance is empirical and must be measured rather than assumed; this is the primary purpose of the qualitative track in Phase 3.

### 5.2 Other limitations

Several further limitations require acknowledgement. First, as a conceptual proposal, the design awaits empirical validation through the staged argument of Section 4; until a corpus exists, its central linguistic claims cannot be measured, and the proposal remains partly aspirational in its NLP requirements for Syrian dialect. Second, the comparative claim underpinning **Tables 1** and **2** is the result of a structured rather than systematic literature review; the mapping covers the multi-agent mental health frameworks identified through the search procedure in Section 2.1, but a formally registered systematic review with reproducible search strings was outside the scope of a Hypothesis and Theory submission and full coverage is not claimed. Priority claims in the abstract, introduction, and conclusion are calibrated accordingly. Third, the mhGAP alignment introduces its own constraints, including documented mental health burden on CHWs delivering psychological interventions (2) and the possibility that mhGAP infrastructure may not be uniformly available across Northwest Syria’s fragmented geography. Fourth, the verification mechanisms described in Section 2.3.6 are themselves model-based and inherit failure modes from their underlying components; we do not claim hallucination is solved, only that it is treated as a first-class design concern with concrete mitigations and an abstention pathway.

The Technical Gap—specifically Arabic NLP readiness—represents the primary implementation bottleneck. This assessment aligns with Fouda et al.’s (13) observation that model selection proves more consequential than language configuration, suggesting that targeted fine-tuning of state-of-the-art multilingual models on even modest Syrian Arabic mental health corpora could yield substantial performance gains. The validation protocol addresses this directly: Phase 1 specifies construction of the SAMHEC corpus through participatory annotation with clinicians and native speakers, establishing the data infrastructure required before any downstream evaluation.

### 5.3 Phased rollout: two architecturally distinct configurations

A consequence of the corpus dependency is that the conversational, free-text screening described in Section 2.3 cannot be the basis of the first version of the system. The two versions are not the same system at two stages of maturity. They differ in the input modality of the Screening Agent, in the meaning of the symptom vector passed to risk stratification, in the clinical interpretability of the stratification thresholds, in how far the verification layer is indispensable, and in the information the human supervisor must hold; these differences are set out in **Table 7**. The distinction also bears on the central hypothesis. Version 1, by relying on structured instruments, largely sidesteps the linguistic-adequacy constraint and so puts only part of the proposition to the test; Version 2, which depends on dialect-aware free-text understanding, is the configuration in which all three constraints — linguistic, cultural, and clinical-responsibility — are simultaneously in play, and is therefore the configuration that would actually test the proposition. This is the deeper reason the two cannot be treated as interchangeable.

**Table 7:** Version 1 and Version 2 as architecturally distinct configurations, not stages of the same system.

Architectural element	Version 1 (pre-SAMHEC)	Version 2 (post-SAMHEC, Phase 1-validated)
Screening Agent input	Structured Arabic-language items (validated PHQ-9, GAD7, and existing Arabic PCL-5 translations where available) administered by a CHW or via a controlled form	Free-text Syrian Arabic conversational input (typed or, in CHW mediated case, ASR-transcribed)
Screening Agent function	Item-response collection and aggregation: a thin layer over standardised instruments	Symptom-signal extraction from open language: a substantive model-based inference, with its own dialectal, code-switching, and ASR uncertainty
Symptom vector s	Direct mapping from structured-instrument item responses to scale-domain scores. Interpretation: actual scale scores.	Probabilistic features reconstructed from text. Interpretation: scale-domain proxies, not scale scores.
Risk Stratification thresholds	PCL-5 ≥ 33, PHQ-9 ≥ 15, GAD-7 ≥ 10 carry the validated clinical meaning of the instruments. Tier assignment is interpretable in standard clinical terms.	The same numerical thresholds cannot be assumed to carry the same meaning, because the inputs are no longer instrument scores. Phase 1 must re-derive tier thresholds against parallel structured-instrument administration before tiers are clinically used.
Verification layer (*K* = 3 resampling; SINdex-style consistency; SAE-style feature monitor)	Optional safety augmentation. Verification is desirable but not architecturally indispensable because the inputs are structured.	Architecturally indispensable. Free-text inputs are the source of the failure modes the verification layer is designed to catch; removing the verification layer in Version 2 would defeat the rationale for permitting free-text input at all.
Human supervisor	Reviews tier assignments and routing decisions; oversees a system whose primary uncertainty is in clinical judgement at the boundary.	Reviews the same outputs *plus* the input-quality and verification flags; oversees a system with an additional, structurally different uncertainty source (model-based language understanding). The supervisor’s information set is larger and the cognitive load is higher.
Status	Does not depend on SAMHEC and could in principle be assembled once partner-NGO infrastructure and ethical approval exist; but it tests only part of the proposition and remains unbuilt and unevaluated.	Presupposes SAMHEC and Phase 1 validation — free-text-to-scale equivalence on the same participants, tier-threshold recalibration, and verification-layer adequacy on Syrian Arabic clinical input — before it could be considered at all.

Differences are not implementation details but changes in the meaning and the failure profile of the corresponding components.

The implication of **Table 7** is that Version 2 is not an upgrade to Version 1; it is a different clinical decision-support configuration that happens to share the routing and follow-up phases. The four-agent decomposition is preserved across versions; the screening–stratification interface, the meaning of the symptom vector, and the role of the verification layer are not. Any deployment claim for the framework therefore needs to specify which version is being claimed, and the validation roadmap (Section 4) treats Phase 1’s free-text-vs-structured comparison as the gate between the two.

## 6 Conclusions

This manuscript has proposed a conditional conceptual-architectural hypothesis for mental health triage in Arabic-speaking post-conflict populations. The theoretical core of the proposal is the claim that AI-assisted triage at a crisis-relevant safety floor in this setting is conditional on the joint satisfaction of linguistic, cultural, and clinical-responsibility constraints (Section 2.2). The four-agent pipeline governed by a Cultural Adaptation Layer, three candidate verification mechanisms, and Human-in-the-Loop oversight is offered as *one possible design response* to that theoretical claim — not as the unique design that satisfies it, and not as a design demonstrated to satisfy it. The preliminary comparative mapping (Section 3) positions this design response relative to published multi-agent mental-health frameworks through a structured, non-systematic procedure, and the validation roadmap (Section 4) is a sequencing argument for future empirical work, not a near-term protocol.

The first necessary step toward any test of the proposal is the construction of the corpus, itself conditional on partnerships and ethical approval that do not yet exist. The two configurations of the system stand in different relations to that step and to the central hypothesis. The structured-instrument configuration does not depend on the corpus and could in principle be assembled earlier, but precisely because it relies on validated instruments rather than on dialect-aware understanding it puts only part of the proposition to the test and should not be mistaken for a realisation of it; nothing here establishes that even this configuration would be safe or effective, and it remains unbuilt and unevaluated. The conversational configuration, which is the one that would test the full proposition, cannot responsibly precede the corpus or the comparison of free-text against structured administration that separates the two.

Because post-traumatic stress is amongst the primary clinical motivations for this work, the status of trauma screening deserves explicit statement: existing Arabic translations of the PCL-5 would be administered as structured instruments where available, but free-text extraction of trauma symptoms is deferred to the conversational configuration and its prior validation, since the concerns that attend reconstructing scale domains from open language apply with particular force to trauma-specific item phrasings. This deferral is a limitation we accept rather than a difficulty we discount; it is reflected in **Table 7**.

The article does not introduce a validated clinical system, and it does not establish that the proposed architecture would be safe or effective in post-conflict Arabic-speaking settings. It advances a theoretical argument about the conditions under which AI-assisted mental-health triage might become clinically interpretable, culturally situated, and institutionally governable in such environments, and offers one candidate architecture as a way of making that argument concrete. The proposal may well require substantial modification, simplification, or partial abandonment in the light of corpus construction, comparative benchmarking, governance review, or clinical evaluation; its value, if it has one, lies in organising the problem and specifying the conditions under which it could be investigated, rather than in offering a solution ready to deploy.

## Data Availability

The original contributions presented in the study are included in the article/supplementary material. Further inquiries can be directed to the corresponding author.
